# Thyroid Hormone Therapy for Potential Heart Donors: A Comprehensive Review of Clinical Trials

**DOI:** 10.3390/biomedicines13071622

**Published:** 2025-07-02

**Authors:** Mushood Ahmed, Eeshal Zulfiqar, Sonia Hurjkaliani, Aimen Shafiq, Hafsa Arshad Azam Raja, Areeba Ahsan, Aemen Kamran, Laveeza Fatima, Amna Nadeem, Muhammad Abdullah Naveed, Faizan Ahmed, Hritvik Jain, Tallal Mushtaq Hashmi, Muath Baniowda, Mansimran Singh Dulay, Sivaram Neppala, Himaja Dutt Chigurupati, Ali Hasan, Peter Collins, Raheel Ahmed

**Affiliations:** 1 Department of Medicine, Rawalpindi Medical University, Rawalpindi 46000, Pakistan; mushood07@gmail.com (M.A.); hafsaazamrajput@outlook.com (H.A.A.R.); tallalhashmi12@gmail.com (T.M.H.); 2 Department of Medicine, Dow University of Health Sciences, Karachi 74200, Pakistan; eeshalzulfiqar12@gmail.com (E.Z.); shurjkaliani@gmail.com (S.H.); aimenshafiq2001@gmail.com (A.S.); a3mee02@gmail.com (A.K.); abdullahnaveed120703@gmail.com (M.A.N.); 3 Department of Medicine, Foundation University Medical College, Islamabad 44000, Pakistan; areebaahsan18@gmail.com; 4 Department of Medicine, Allama Iqbal Medical College, Lahore 05450, Pakistan; laveezaghafoor2002@gmail.com; 5 Department of Medicine, Faisalabad Medical University, Faisalabad 37000, Pakistan; nadeemamna116@gmail.com; 6 Department of Cardiology, Duke University Hospital, Durham, NC 27247, USA; drfaizanamalik@gmail.com; 7 Department of Medicine, All India Institute of Medical Sciences, Jodhpur 112001, India; hritvikjain2001@gmail.com; 8 Department of Medicine, University of Missouri, Kansas City, MO 63053, USA; muathbaniowda@hotmail.com; 9 Department of Cardiology, Royal Brompton Hospital, London SW3 6NP, UK; m.singhdulay@rbht.nhs.uk; 10 Department of Cardiology, Kings College, London WC2R 2LS, UK; 11 Division of Cardiology, UT Health Science Center, San Antonio, TX 78229, USA; sivaram894@gmail.com; 12 Division of Cardiology, East Carolina University, Greenville, NC 27858, USA; himajadutt.chigurupati@gmail.com; 13 Department of Cardiology, Imperial College, London SW7 2AZ, UK; ali.hasan21@imperial.ac.uk (A.H.); peter.collins@imperial.ac.uk (P.C.); 14 Department of Cardiology, National Heart and Lung Institute, Imperial College, London SW7 2AZ, UK

**Keywords:** thyroid hormone, triiodothyronine, tetraiodothyronine, heart donors

## Abstract

**Background:** Due to neurohormonal disturbances that occur following brain death, thyroid hormone therapy has been proposed as a means to enhance cardiac function in brain-dead organ donors. However, it remains unclear whether thyroid hormone administration improves clinical outcomes in potential heart donors. **Methods:** A comprehensive review of clinical trials was conducted to evaluate the impact of thyroid hormone therapy on heart viability and transplantation outcomes. A total of nine randomized controlled trials (RCTs) involving 1189 potential heart donors were included. **Results**: Thyroid hormone supplementation effectively restored circulating thyroid hormone levels in brain-dead donors. However, findings regarding improvements in cardiac function and transplantation outcomes were inconsistent across studies. While some RCTs reported marginal improvements in hemodynamic parameters and heart transplant viability, these results were not consistently replicated. Furthermore, most studies did not demonstrate a significant enhancement in recipient survival or graft function associated with thyroid hormone therapy. **Conclusion:** Although thyroid hormone therapy restores thyroid hormone levels in brain-dead donors, current evidence does not consistently support its effectiveness in improving donor heart viability or recipient outcomes. Further research is necessary to clarify the role of thyroid hormone therapy in donor management and its impact on long-term transplant success.

## 1. Introduction

Heart transplantation remains an important treatment for patients with advanced heart failure [1]. The vast majority of transplanted hearts are from donors following brain death (DBD) [1]. However, less than half of donor hearts are considered eligible for transplantation because brain death commonly causes systemic derangements that severely impact organ donation [2,3]. Hence, there is an increased need for more sophisticated donor management to enhance organ quality and availability.

Brain death is marked by a malfunctioning neurohormonal axis and an autonomic storm, along with a sharp drop in hormones like cortisol, insulin, thyroxine (T4), triiodothyronine (T3), and antidiuretic hormone [4,5]. Neurohormonal insufficiency, particularly a thyroid hormone deficit, causes myocardial energy loss and shock following brain death [6]. This cascade of physiological changes associated with brain death adversely affects the cardiovascular system, often leading to hemodynamic instability, myocardial dysfunction, and eventually the loss of potential donor organs [7].

Thyroid hormones, particularly T3, exert both genomic and non-genomic effects on cardiac myocytes and vascular smooth muscle. Genomically, T3 regulates the expression of key proteins, such as myosin heavy chains and sarcoplasmic reticulum Ca^2+^-ATPase (SERCA), while downregulating phospholamban, enhancing calcium handling and myocardial contractility [8]. Non-genomically, T3 increases phosphorylation of phospholamban via kinase pathways, promoting diastolic relaxation [8]. Thyroid hormones also upregulate β-adrenergic receptors, augmenting inotropic and chronotropic responsiveness, and promote vasodilation through enhanced endothelial nitric oxide production and direct smooth muscle relaxation, reducing systemic vascular resistance [9].

Given the role of thyroid hormones in modulating cardiac contractility, vascular tone, and metabolism, amelioration using thyroid hormone therapy could be an effective approach to counter some of these harmful effects. Guidelines for donor management recognize the use of donor hormonal therapy to increase the number of organs that can be transplanted [10]; studies with long-term follow-up have shown that thyroid hormone supplementation is helpful [11]. Several studies have demonstrated that thyroid hormone treatment induces donor heart contractility, supports the hemodynamic profile, and helps expand the suitable donor pool for transplantation. Nevertheless, the consensus of evidence is mixed, with some studies demonstrating significant advantages and others reporting no clear benefit. This review aims to evaluate the published clinical trial data on the effectiveness and safety of thyroid hormone therapy in DBD donors, taking into account the variability in existing data. We aim to source these studies and narratively synthesize their data to provide a comprehensive assessment of the effects of the prescription of thyroid hormone supplementation on outcomes of cardiac donation and transplantation.

## 2. Methods

The study was conducted following Preferred Reporting Items for Systematic reviews and Meta-Analyses guidelines [12] and the protocol of review was registered with PROSPERO (CRD420251071186).

### 2.1. Data Source and Search Strategy

The literature search involved multiple major databases, including PubMed, Scopus, Cochrane Library, and Web of Science, to include all relevant studies published from their inception to August 2024. The search strategy used a combination of key terms, such as “thyroid hormone therapy”, “brain-dead donors”, “cardiac donors”, “organ donation”, “thyroid hormone replacement”, and specific hormones such as “T3”, “T4”, and “levothyroxine”. Additionally, terms related to clinical outcomes such as “efficacy”, “safety”, “organ function”, “cardiac output”, and “outcomes” were included. Boolean operators and Medical Subject Headings (MeSH) were applied to refine the search process, ensuring comprehensive coverage of the available literature. No language restrictions were applied. The search strings used for each database along with the retrieved results are provided in Table S1.

### 2.2. Eligibility Criteria and Outcomes

Two independent reviewers screened the titles and abstracts of all the retrieved studies to determine eligibility based on predefined inclusion and exclusion criteria. Studies were included if they (1) involved DBD donors, (2) reported the use of thyroid hormone therapy, (3) provided data on clinical outcomes including transplantation rates, 30-day graft survival, hemodynamic status, and cardiac function, and (4) were randomized controlled trials (RCTs) or their reports. Exclusion criteria included studies without control groups, animal studies, and case reports. Disagreements between reviewers were resolved through discussion, or by consulting a third reviewer when necessary.

### 2.3. Study Selection and Data Extraction

Following a thorough search, the identified studies were imported into Mendeley Desktop 2.112.0 (Mendeley Ltd., Amsterdam, The Netherlands) for further screening. Duplicate articles were identified and subsequently removed. To ensure accuracy, two authors conducted a review of the remaining articles and exclusively selected those studies that met our outlined eligibility criteria. In case of any discrepancies concerning study selection, a senior author was consulted to aid in discussions to find a resolution.

To ensure consistency in data collection, two independent authors extracted the data into an Excel spreadsheet that was pre-piloted. We extracted the following data from each eligible study: study design, country, thyroid therapy, sample size, age, years, males, and main findings. A third author reviewed the extracted data to ensure accuracy.

### 2.4. Bias Assessment

Two reviewers independently performed a systematic assessment of the included studies. The risk of bias was evaluated using the RoB 2 tool, a revised method for assessing bias in randomized trials [13]. Bias was assessed across five key domains: randomization, deviations from the intended interventions, missing outcome data, outcome measurement, and selection of reported results. Each trial was rated as having high risk, some concerns, or low risk of bias within each domain. Risk-of-bias plots were created using the Robvis tool [14].

## 3. Results

**Figure 1:**
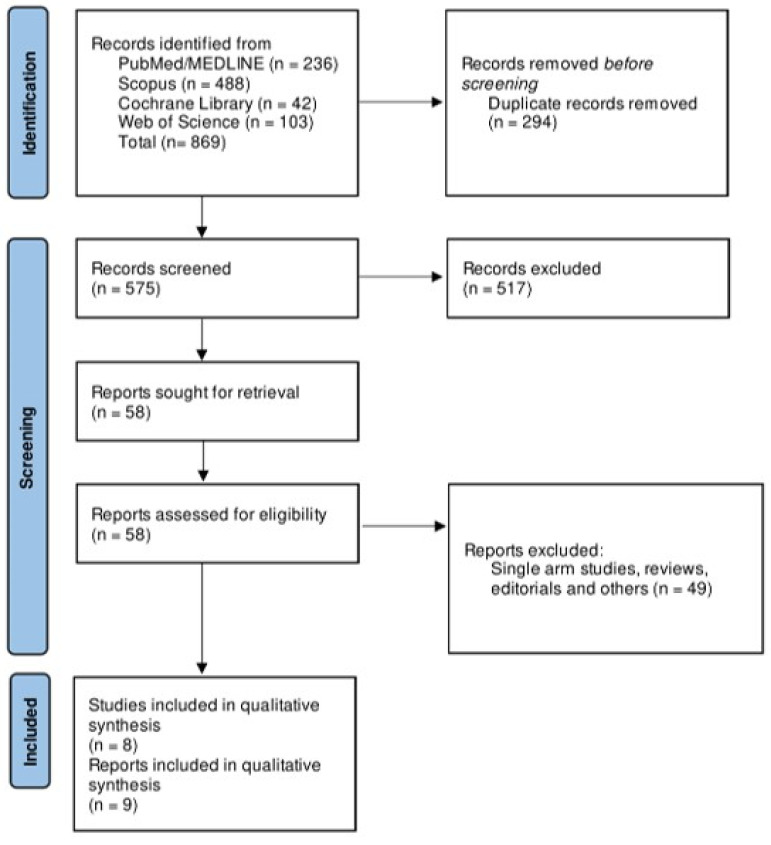
Flowchart showing the screening and study selection process.

**Figure 2:**
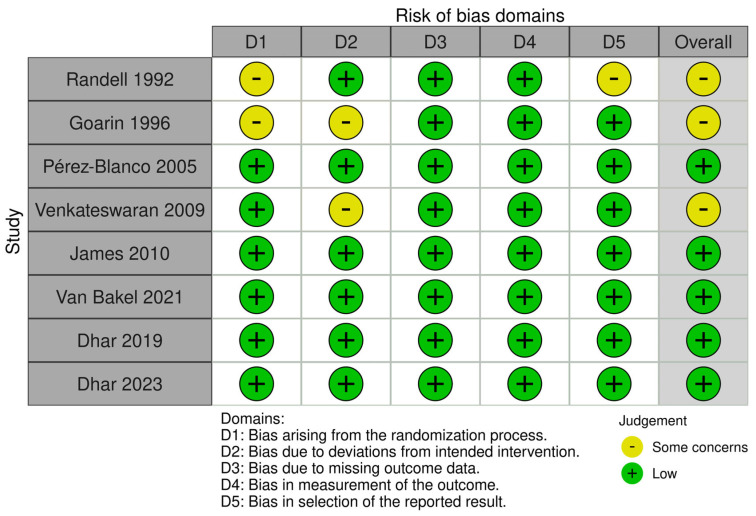
Risk of bias assessment for included RCTs [15,16,17,18,19,20,21,22].

The literature search yielded a total of 869 records. Duplicate articles were excluded, and records were screened by reviewing their titles and abstracts. A total of 517 studies were excluded after primary screening, and full texts of the remaining 58 articles were assessed. A total of nine reports reporting data for eight RCTs that fulfilled the predetermined inclusion criteria were included in our systematic review. The details of the study screening are shown in Figure 1. A meta-analysis was not conducted due to the considerable between-study heterogeneity and differences in treatment protocols. The bias assessment of included studies showed some concerns in three RCTs [15,16,17], mainly related to the randomization process, deviations from intended intervention, and reporting of results. The details of the bias assessment are reported in Figure 2.

In this section, we summarize the findings of all known trials so far on thyroid hormone therapy among heart donors. Individual study characteristics are given in Table 1, with details of other interventions in Table S2.

### 3.1. Randell et al. (1992): Impact of Thyroid Hormone Therapy on Hemodynamic Stability in Multiorgan Donors [15]

Randell et al. [15] reported a randomized controlled study in 1992 that aimed to assess the effect of thyroid hormone therapy on hemodynamic stability and temperature maintenance on DBD, multiorgan donors. The study involved 25 donors, divided into a T3 group (n = 13) and a control group (n = 12). The findings indicate no statistically significant differences in hemodynamic parameters, including heart rate and arterial blood pressure, between the two groups. The duration of surgery was longer in the control group compared to the treatment group (198 ± 34 vs. 171 ± 27 min, respectively). Other outcomes measured in the study included liver function, rate of dopamine infusion, duration of hospital stay, and intubation. Sodium chloride and blood glucose levels were also monitored, though no significant differences were observed between the groups in terms of blood gases, perfusion, or fluid administration.

### 3.2. Goarin et al. (1996): Effects of Triiodothyronine on Hemodynamic and Echocardiographic Parameters in Heart Donors [16]

In 1996, Goarin et al. [16] reported a prospective, randomized, blinded, placebo-controlled study of 37 DBD donors in France; 19 were administered triiodothyronine while 18 received a placebo. The study aimed to evaluate the effect of T3 administration on hemodynamic and echocardiographic parameters in potential heart donors. While T3 administration successfully normalized serum T3 levels (7.55 ± 2.56 pmol/L compared to 1.48 ± 1.26 pmol/L in controls), this did not result in improved cardiac function. No significant differences were observed in hemodynamic status, left ventricular function, preload, afterload, or ejection phase indices in either group, including those with impaired left ventricular function (defined as fractional area change [FAC] < 50%). Moreover, there was no correlation between baseline T3 levels and cardiac function, suggesting that low T3 concentrations were not a significant determinant of myocardial dysfunction in brain-dead donors.

### 3.3. Pérez-Blanco et al. (1997–2000): Triiodothyronine and Its Impact on Hemodynamics and Oxygen Utilization in Cardiac Donors [18]

Between May 1997 and May 2000, Pérez-Blanco et al. [18] conducted a prospective, randomized, double-blinded, placebo-controlled study to compare the effects of triiodothyronine (T3) treatment versus standard support on hemodynamic function, oxygen utilization, and adenine nucleotide concentrations in brain-dead cardiac donors. A total of 52 DBD donors were included, with 29 allocated to the treatment group and 23 to the placebo group. The eligibility criteria involved a confirmed diagnosis of brain death, suitability for organ transplantation, and family written informed consent for donation. The exclusion criteria were limited to donors with preexisting thyroid disease. Hemodynamic variables, serum lactate levels, and biopsied organ adenine nucleotide concentrations were monitored every 90 min from brain death until organ procurement. No significant differences in hemodynamics or adenine nucleotide concentrations were observed between the two groups. There was no statistically significant difference in cardiac index (CI) between the treatment and control groups. For instance, at t270, CI was 4.4 ± 2.2 in the treatment group compared to 4.7 ± 1.7 in the control group. Similarly, the results were comparable for oxygen extraction (O2E%) and Pco2 Gap. Serum lactate levels were significantly lower in the treatment group compared to the controls.

### 3.4. Venkateswaran et al. (2004–2006): Cytokine Levels and Donor Organ Suitability in Response to Thyroid and Methylprednisolone Therapy [17]

A prospective randomized double-blind trial, conducted from January 2004 to April 2006 by Venkateswaran et al. [17], evaluated cytokine levels (IL-1, IL-6, TNF-α, CRP, and PCT) as a secondary outcome in heart and lung donors aged 16–65 years. Donors were randomized to receive T3, methylprednisolone (MP), both, or a placebo after a comprehensive cardiopulmonary assessment. The study aimed to optimize donor organ function and transplantation suitability, with hemodynamic and lung management closely monitored. While final cytokine levels showed no significant differences between treatment groups, initial PCT levels correlated with cardiac function; donors with PCT > 2 ng·mL−1 had worse cardiac indices (CI) (0.11± 0.6), lower left ventricular ejection fraction (LVEF) (53.8 ± 19.3), and right ventricular ejection fraction (RVEF) (20.8 ± 7.3). However, 5 of 17 hearts with elevated PCT levels were still suitable for transplantation. Ultimately, 40 of 79 hearts (51%) and 60 of 120 lungs (50%) met the transplant criteria, achieving a 30-day survival rate of 96% for both organs. These results suggest that baseline PCT levels may be a useful predictor of heart transplant suitability, although their broader role in donor management requires further research.

### 3.5. Venkateswaran et al. (2004–2006): Influence of Thyroid and Methylprednisolone Therapy on Donor Heart Function [23]

The effect of methylprednisolone (MP) and thyroid hormone therapy (T3) on donor heart function and transplant outcomes was examined by Venkateswaran RV et al. [23], between January 2004 and April 2006. Eighty cardiac donors, aged sixteen to sixty-five, were split into four groups at random: T3, MP, T3 plus MP, and placebo. The MP group received a 1000 mg IV bolus, while the T3 group received an IV bolus of 0.8 µg/kg followed by a 0.113 µg/kg/h infusion. With the use of sophisticated catheters, hemodynamic measures such as the cardiac index (CI), systemic vascular resistance (SVR), and the stroke work index (SWI) were tracked.

T3 therapy increased serum T3 levels, but donor heart function did not significantly improve as a result. Both the CI and SVR dropped from 1190 ± 572 to 964 ± 381 dyne cm s^−5^ and 3.18 ± 1.1 to 3.72 ± 1.5 L/min/m^2^, respectively, although these differences were not statistically significant between the therapy groups. Of the 50% of the hearts that were considered viable for transplantation, 31% had successful transplantation, yielding a 96% 30-day survival rate. Thyroid hormone therapy appears to have little value in improving the results of donor heart transplants, since it did not significantly influence recipient survival or graft acceptance.

### 3.6. James et al. (2010): Hemodynamic Response to Triiodothyronine in Brain-Dead Cardiac Donors [19]

James et al. [19] also published a prospective double-blinded, placebo-controlled trial in 2010, involving 30 brain-dead cardiac donors, randomized to either T3 (n = 16) or a placebo (n = 14). Informed consent was obtained from the family of the donor. T3 administration successfully reversed the low fT3 state, increasing serum fT3 levels from 3.21 (2.30–4.13) at baseline to 14.39 (12.13–16.74) in the treatment group by the end assessment (*p* < 0.001). Hemodynamic parameters, such as central venous pressure (CVP), pulmonary capillary wedge pressure (PCWP), and CI, increased similarly in both groups from baseline to pre-retrieval, with the systemic vascular resistance index (SVRI) decreasing; however, these findings did not reach statistical significance. The LV stroke work index (LVSWI) and cardiac power output index (CPOi) were also comparable between the two groups.

### 3.7. Van Bakel et al. (2010–2012): Outcomes of Thyroid Hormone Therapy in Adult Organ Donors [20]

The study by Van Bakel AB et al. [20] investigated the effects of thyroid hormone therapy (T4) on adult organ donors from September 2010 to August 2012, examining outcomes like vasopressor requirements, thyroid hormone levels, and organ recovery. Donors aged 18–70 were randomized into four groups: Levo (T4 infusion), MP (methylprednisolone), Combo (T4 + MP), and Control (no T4 or MP). Standard donor management protocols were followed, including volume resuscitation, hemodynamic monitoring, and vasopressor titration. Methotrexate (high dose) was administered to the MP group, and T4 (titrated up to 200 µg/h) was given to the Levo group. The vasopressor–inotropic score (VIS) was used to quantify the need for vasopressors.

The Levo and Combo groups’ total and free T3 levels were considerably raised by thyroid hormone therapy; the percentage of donors with low free T3 levels fell from 72.6% to 10.6% in the Combo group and from 61.1% to 5.9% in the Levo group. Except for the Levo group, all the groups saw a drop in VIS despite improvements in thyroid hormone levels; no discernible changes in cardiac index were seen. Organ-specific outcomes revealed that of the 1272 organs consented for procurement, 725 (57%) were recovered, yielding 3.64 organs per donor. A total of 616 organs were transplanted (85% of recovered organs), with a high recipient outcome reporting rate of 90.3%. Graft survival and patient outcomes were consistent across all treatment groups, with no significant differences in graft acceptance or survival observed.

### 3.8. Dhar et al. (2015–2017): Impact of T4 Therapy on Left Ventricular Ejection Fraction in Brain-Dead Donors [21]

Dhar et al. [21] assessed 77 brain-dead organ donors, in a single OPO recovery facility in a prospective randomized experiment that took place between January 2015 and June 2017. Among the donors with a left ventricular ejection fraction (LVEF) of less than 60%, eight hours of T4 infusion (median baseline LVEF: 45%, IQR 42.5–47.5) or placebo infusion (median baseline LVEF: 40%, IQR 40–50) was randomly assigned. Improvement in LVEF was the main result; rates of heart transplants and adjustments to thyroid hormone levels were the secondary outcomes. According to the results, the T4 group’s median LVEF improvement was 10% (IQR 5–15), while the control group’s was only 5% (IQR 0–12.5). LVEF ≥ 60% was attained by 60% of the T4-treated donors, against 11% in the control group (*p* = 0.03). Additionally, there was a trend towards more hearts being transplanted (59% vs. 27%, *p* = 0.14) and a larger final cardiac stroke volume (88 mL vs. 57 mL, *p* = 0.02) in the T4 group. Furthermore, a history of illegal drug use was linked to higher rates of heart transplantation (63% vs. 11%, *p* = 0.016) and larger LVEF improvement (median 15%, IQR 5–15, *p* = 0.01). Thyroid hormone levels showed little change, with the T4 group showing a small increase in fT4 levels (0.83–0.92, *p* = 0.02), indicating that more study is required on T4 infusion in donor management.

### 3.9. Dhar R et al. (2020–2022): Levothyroxine Versus Saline in Deceased Organ Donors: A Multicenter Trial [22]

A multicenter, parallel-group, randomized trial by Dhar R et al. [22] investigated the effects of levothyroxine versus saline in deceased organ donors across 15 organ procurement organizations (OPOs) in the United States between 1 December 2020 and 6 November 2022. Eight hundred and fifty-two brain-dead donors, aged 14 to 55, who fulfilled the neurological requirements for death and needed inotropes or vasopressors were included in the study. Heart transplantation was the key efficacy endpoint, while 30-day graft survival was the main safety outcome. Out of the 838 donors examined, transplanted hearts were received by 54.9% of the levothyroxine group and 53.2% of the saline group, with 30-day graft survival rates of 97.4% and 95.5%, respectively. There was no significant difference between the two groups’ 30-day graft survival rate or heart transplantation rates, even though the levothyroxine group’s free T4 levels increased by 37%. Levothyroxine treatment was linked to a higher incidence of adverse events, such as hypertension and tachycardia, which resulted in the early discontinuation of the infusion in 21% of cases. It also did not improve left ventricular ejection fraction (LVEF) or reduce the need for vasopressors. Levothyroxine raised thyroid hormone levels overall, but there was no discernible improvement in transplantation results.

### 3.10. Summary of Clinical Trial Findings

1. Hemodynamic and Cardiac Function

Thyroid hormone therapy (primarily T3) was used in several RCTs for its impact on heart donor hemodynamics, but the results showed no significant improvements in cardiac indices or left ventricular function.

2. Heart Transplant Rates

While some studies suggested that thyroid hormone therapy may stabilize hemodynamic parameters, there was minimal evidence of enhanced transplant suitability or success rates for donor hearts.

3. Serum Thyroid Hormone Levels

Thyroid hormone treatment increased serum T3 or T4 levels in donors; however, elevated hormone levels did not consistently lead to improved donor heart function or transplant outcomes.

4. Transplant and Survival Rates

The studies observed no statistically significant improvement in heart transplant or 30-day survival rates among recipients, indicating a limited benefit from thyroid hormone therapy in improving heart donor outcomes.

5. Vasopressor Needs

Thyroid hormone therapy reduced vasopressor requirements in some studies; yet this did not translate to an improvement in transplant success, leaving its role in enhancing donor heart viability unclear.

## 4. Discussion

In this review, we sought to evaluate whether thyroid hormone therapy in DBD donors improves transplantation rates, 30-day graft survival, hemodynamic status, and cardiac function. Most of the studies included in our review failed to demonstrate statistically significant differences in these key outcomes when comparing thyroid hormone therapy to placebo or control groups. While some studies observed minor improvements in hemodynamic stability with thyroid hormone administration, these findings were inconsistent across trials, suggesting that the benefits of thyroid hormone therapy in donor management remain uncertain.

Van Bakel et al. [20] conducted intention-to-treat (ITT) and per-protocol (PP) analyses to determine whether early administration of T4, methylprednisolone (MP), or both could enhance hemodynamic stability by reducing vasopressor needs. This suggests that corticosteroids, particularly when used in higher doses, may exert a more substantial influence on hemodynamic stability than TH alone. These results contrast with the findings of a blinded, randomized trial by Venkateswaran et al. [23], which showed no improvement in cardiac index (CI) with T3, MP, or their combination compared to controls. Key differences in their study, including a lower dose of MP and shorter treatment duration, may have limited the potential benefits. Additionally, they did not quantify vasopressor agents, further complicating the assessment of treatment effects on CI.

Moreover, two recent publications by Peled et al. [24,25] provide a cautionary note against thyroid hormone use, citing higher incidences of primary graft dysfunction in treated hearts. They suggest that β-adrenergicreceptor upregulation and diminished mitochondrial energy stores may contribute to an adverse TH withdrawal effect, complicating outcomes in TH-treated donors. This aligns with findings from Dhar et al. [22], who observed slightly higher 30-day graft survival rates in the levothyroxine-treated group compared to the saline group; however, the difference was not significant. Additionally, there were no substantial differences in survival between subgroups defined by various factors, such as the timing of infusion, LVEF, baseline free T4 levels, vasopressor dose, or donor cause of death. Notably, the study also observed a higher frequency of adverse events, including hypertension and tachycardia, in donors receiving levothyroxine, although these issues resolved upon stopping the infusion.

Donor management protocols in most of the included trials employed various adjunct interventions aimed at improving donor cardiac function and stability (Table S2). These included corticosteroids (most commonly MP or hydrocortisone), vasopressin for the management of diabetes insipidus, catecholamines such as dopamine and norepinephrine for hemodynamic support, and volume resuscitation strategies using crystalloids or colloids. Some studies also included insulin–glucose infusions to modulate metabolic parameters and reduce ischemic risk [20,23]. Among these, corticosteroids were the most consistently used alongside TH and have shown some independent benefit in reducing vasopressor requirements. While the thyroid hormone was frequently used as part of a broader hormonal resuscitation strategy, its relative contribution remains debated. It often served as a complementary agent, particularly in donors already optimized with fluids and vasopressors. However, the lack of standardized regimens and frequent co-administration of other agents makes it difficult to isolate the specific impact of TH in most trials.

Improvement in both the quality and quantity of organs for transplantation is the ultimate goal of donor care [26,27]. This is a challenge in mitigating the physiological disturbances that occur in the context of brain death and contribute to cardiac dysfunction and hemodynamic instability [28,29]. It was for the hemodynamic stabilization of brain-dead organ donors that hormonal therapy was initiated by cardiothoracic surgeons at the University of Cape Town in the early 1980s, after it had been noted with concern that roughly 20% of the hearts from brain-dead donors were unsuitable for transplantation due to poor myocardial function [30]. The importance of donor management henceforth required hormone resuscitation therapy (HRT) HRT to play an essential role, and this has been mainly carried out by the administration of TH in addition to corticosteroids to enhance organ viability and stabilize hemodynamics.

However, few randomized trials assessing thyroid hormone administration in donors were adequately powered to assess organ utilization, focusing instead on intermediate outcomes such as donor hemodynamics [16,18,20,23]. Despite the paucity of high-quality evidence supporting this approach, consensus recommendations still advise thyroid hormone use in hemodynamically unstable or heart-eligible donors [10,27,31]. Nevertheless, concerns remain, as some research has suggested that early allograft failure rates may be higher with thyroid therapy [24,25].

Conflicting evidence from observational and randomized studies has led to inconsistent findings in recent meta-analyses [31,32,33]. Variations in treatment plans, dosage, administration schedules, and study endpoints have all contributed to these inconsistent results. While some donors may respond favorably to volume loading, many still require vasopressor support to maintain sufficient perfusion pressure to target organs. Severe vasoconstriction, particularly in response to high-dose catecholamines like norepinephrine, has been observed and may be detrimental to potentially transplantable hearts. Conversely, lower-dose dopamine support might be beneficial for heart and renal function [34,35,36,37]. However, a recent retrospective study demonstrated no difference in one-year mortality among heart recipients from donors supported with no, low, or high doses of norepinephrine [38]. The authors of that study noted that their findings were primarily applicable to selected donor–recipient sex combinations with anticipated short ischemic times. Overall, maintaining hemodynamic stability while minimizing catecholamine vasopressor use remains a critical objective in the management of deceased organ donors.

While most studies included in our review demonstrated no significant benefit of thyroid hormone therapy on hemodynamic stability and cardiac function, this could be attributed to their small sample sizes, limiting their ability to detect important clinical effects [15,16,18,19]. Unlike the smaller clinical trials, a large retrospective analysis from the UNOS database, which evaluated 63,593 brain-dead organ donors over a 10-year period, reported a significant 12.8% increase in organ procurement rates with thyroid hormone supplementation [39]. However, this benefit did not translate into improved graft survival, aligning with the results observed in our review.

This study has several limitations. The majority of the included studies had small sample sizes, which limits the generalizability of our findings. Additionally, the studies span over several decades, from 1992 to 2023, which may result in variations in medical practices and donor management protocols that could influence the comparability of results. Moreover, variations in the thyroid hormone therapies used across studies (e.g., triiodothyronine, levothyroxine, thyroxine) introduce heterogeneity, making direct comparisons of outcomes more difficult. Furthermore, potential publication bias cannot be ruled out, as studies with negative or inconclusive results may have been less likely to be published. Finally, variability in how transplant suitability and organ acceptance were reported across centers and time periods introduces an additional layer of inconsistency, which may affect the interpretation of thyroid hormone therapy’s effectiveness in improving donor organ viability.

## 5. Conclusions

In conclusion, our review suggests that thyroid hormone therapy in DBD donors shows non-significant improvements in transplantation rates, graft survival, and hemodynamic stability. Future research should prioritize large-scale randomized trials with longer follow-up durations and standardized protocols to clarify the role of thyroid hormone therapy in donor management. Exploring the long-term effects on graft function and survival, as well as potential adverse outcomes, will be critical to optimizing donor care strategies.

## Figures and Tables

**Table 1:** Baseline characteristics of included studies and their main findings.

Study	Study Design	Country	Thyroid Therapy	Sample Size (N)		Age, Years (Mean ± SD)		Males (n)		Main Findings
				Thyroid Therapy	Control	Thyroid Therapy	Control	Thyroid Therapy	Control	
Randell 1992 [15]	RCT	Finland	Triiodothyronine (T3)	13	12	33 ± 13	34 ± 11	6	6	No statistically significant differences in hemodynamic parameters.
Goarin 1996 [16]	RCT	France	Triiodothyronine (T3)	19	18	35 ±10	34 ± 14	11	13	No statistically significant differences in hemodynamic status or cardiac function.
Pérez-Blanco 2005 [18]	RCT	Spain	Triiodothyronine (T3)	29	23	38.4 ± 17	40.7 ± 23.3	-	-	No statistically significant differences in hemodynamic status or cardiac index.
Venkateswaran 2009 [17]	RCT	UK	Triiodothyronine (T3)	20	20	-	-	-	-	51% of hearts and 50% of the lungs met the transplant criteria, achieving a 30-day survival rate of 96% for both organs.
Venkateswaran 2009 [23]	RCT	UK	Triiodothyronine (T3)	20	21	-	-	-	-	No significant improvements in donor heart function; Of the 50% of hearts that were considered viable for transplantation, 31% had successful transplantation, yielding a 96% 30-day survival rate.
James 2010 [19]	RCT	UK	Triiodothyronine (T3)	16	14	48.03 ± 9.84	49 ± 10.54	-	-	No statistically significant differences in hemodynamic parameters.
Van Bakel 2021 [20]	RCT	USA	Levothyroxine (T4)	49	49	43.3 ± 13.6	38.7 ± 13.4	29	35	No statistically significant differences in hemodynamic status or cardiac index; Graft survival and patient outcomes were consistent across all treatment groups; A total of 616 organs were transplanted (85% of recovered organs).
Dhar 2019 [21]	RCT	USA	Thyroxine (T4)	17	11	29.2 ± 8.3	30.7 ± 9.5	9	6	Significant improvements were seen with T4 infusion; 60% of T4-treated donors achieved an LVEF ≥ 60% compared to 11% in the control group (*p* = 0.03). The T4 group had a median LVEF improvement of 10% versus 5% in controls (*p* = 0.03); larger cardiac stroke volume (88 mL vs. 57 mL, *p* = 0.02). No significant difference in heart transplantation rates (*p* = 0.14).
Dhar 2023 [22]	RCT	USA	Levothyroxine (T4)	419	419	36 ± 11	36 ± 10	276	247	No significant differences in 30-day graft survival rate or heart transplantation rates; Levothyroxine treatment was linked to a higher incidence of adverse events.

n = number, RCT = randomized controlled trial.

## Data Availability

The data that support the findings of this study are available from the corresponding author upon reasonable request.

## Supplementary Materials

The following supporting information can be downloaded at https://www.mdpi.com/article/10.3390/biomedicines13071622/s1, Table S1: Detailed search strings used for various databases with records retrieved; Table S2: Details of other interventions used in the included studies.
