# Supplementary material for: Thyroid Hormone Therapy for Potential Heart Donors: A Comprehensive Review of Clinical Trials
Source: Biomedicines. 2025 Jul 2;13(7):1622. doi: 10.3390/biomedicines13071622 (PMC12293070; doi:10.3390/biomedicines13071622)
Supplement: Supplementary file 1 [file biomedicines-13-01622-s001.zip › biomedicines-3719311-supplementary.pdf]

Supplementary Appendix

Table S1: Detailed search strings used for various databases with records retrieved

Database	Search String	Records
PubMed/MEDLINE	("Thyroid Hormones"[All Fields] OR "thyroid hormone"[All Fields] OR "triiodothyronine"[All Fields] OR "thyroxine"[All Fields] OR "levothyroxine"[All Fields] OR "T3"[All Fields] OR "T4"[All Fields]) AND ("Heart Donors"[All Fields] OR "Organ Donors"[All Fields] OR "heart donor"[All Fields] OR "organ donation"[All Fields] OR "cardiac donation"[All Fields] OR "heart transplantation"[All Fields])	236
Scopus	(TITLE-ABS-KEY("thyroid hormone" OR "triiodothyronine" OR "thyroxine" OR "levothyroxine" OR "T3" OR "T4")) AND (TITLE-ABS-KEY("heart donor" OR "heart donors" OR "organ donors" OR "heart transplantation" OR "cardiac donation"))	488
Web of Science	TS=("thyroid hormone" OR "triiodothyronine" OR "thyroxine" OR "levothyroxine" OR "T3" OR "T4") AND TS=("heart donor" OR "heart donors" OR "organ donors" OR "heart transplantation" OR "cardiac donation")	103
Cochrane Library	("thyroid hormone" OR "triiodothyronine" OR "thyroxine" OR "levothyroxine" OR "T3" OR "T4") AND ("heart donor" OR "heart donors" OR "organ donors" OR "heart transplantation" OR "cardiac donation")	42

Table S2: Details of Other interventions used in the included studies

Study	Other interventions used
**Randell et al. (1992) [13]**	Hydroxyethyl starch, Ringer's acetate, 0.45% NaCl, dopamine (as needed), calcium chloride, mannitol, desmopressin, mechanical ventilation, warmed IV fluids
**Goarin et al. (1996) [14]**	Colloid preload (for hypovolemia), crystalloids per protocol, dopamine infusion to maintain MAP >65 mmHg, mechanical ventilation
**Pérez-Blanco 2005 [16]**	Crystalloids, red blood cell transfusion, dopamine (2–8 μg/kg/min), desmopressin, famotidine, ventilator support, maintenance of core temperature
**Venkateswaran 2009 [15]**	Methylprednisolone (MP), vasopressin, weaning off norepinephrine, fluid limitation, colloid transfusion, mechanical ventilation with alveolar recruitment, inotropes (as needed), hemodynamic optimization with CI/PCWP targets
**Venkateswaran 2009 [17]**	Methylprednisolone, vasopressin, weaning of norepinephrine/inotropes, limited blood or colloid transfusion, insulin infusion, mechanical ventilation, pulmonary artery catheter–guided hemodynamic optimization (CI, MAP, PCWP, SVR targets)
**James 2010 [18]**	Gelatin-based colloids, vasopressin (as needed), withdrawal of norepinephrine and inotropes, hemodynamic targets via pulmonary artery catheter monitoring

**Van Bakel 2021 [19]**	Methylprednisolone (30 mg/kg + repeat dose), vasopressin for DI, insulin + glucose, blood transfusions (Hb >10 g/dL), fluid resuscitation, vasoactive drugs, pulmonary artery catheter-guided management
**Dhar 2019 [20]**	Standardized goal-directed fluid resuscitation (NS boluses), vasopressor weaning, vasopressin for DI, IV hydrocortisone (300 mg loading + 100 mg q8h), mechanical ventilation
**Dhar 2023 [21]**	Usual donor care per OPO policy (non-standardized), with optional continuation or open-label levothyroxine use post-infusion
